# Among the Honduranians, Ancient and Modern

**Published:** 1902-05

**Authors:** George Byron Gordon

**Affiliations:** Cambridge, Mass.


### AMONG THE HONDURANIANS, ANCIENT AND MODERN.[1]

BY GEORGE BYRON GORDON, CAMBRIDGE, MASS.

MR. PRESIDENT, AND FELLOWS OF THE ACADEMY,—The subject on which I have the honor of addressing you is, as the President has just said, Honduranians, Ancient and Modern; and I may say that of these two, the ancient Honduranian and the modern Honduranian, it is the ancient Honduranian that appeals more strongly to my sympathies and has the greater claim upon my imagination. It is very likely that the modern Honduranian might appeal more strongly to our sense of humor; but in all serious aspects of scientific research, in all that concerns the student of human affairs, of political organization and social evolution, of the development of arts and sciences, it is the ancient Honduranian alone with whom we are concerned.

To speak correctly, the ancient Honduranian is not a Honduranian at all. He is a member, or was a member, of a great nation which dominated all of Central America, from Tehuantepec to Darien; whereas the name Honduras, applied first by the Spaniards at the time of the Conquest to that part of the main-land which borders on the Caribbean Sea, is applied to-day to the political division of Honduras, one of the five little republics into which Central America is divided. The name means the land of great depths, the land of mystery, the land of profound solitude. All of these things the name "Honduras" means in the Spanish tongue, and it is of especial significance, showing as it does that at the time when the Spaniards first arrived there, of all lands they had seen it was the one that most impressed them

---

[1] Read before the American Academy of Dental Science, January, 1902.

with the sense of solitude. And yet—although they did not know it—those same forests, before which the intruders paused in dismay, hid the ruined temples and palaces of a people much more powerful, much greater, and possessed of a vaster wealth than Cortez or Pizarro ever saw. I dwell particularly on this point, because it has been stated on what is considered high authority that the Spaniards actually came in contact with the civilization of the ancient people of Central America, and it was through the agency of the conquerors that that civilization was destroyed. This is not so. Whatever fate may have befallen them, that fate was not the fate of Mexico and Peru. The strange, mysterious race that at one time reclaimed this part of the American Continent from the wilderness, built upon its beautiful and fertile valleys immense cities, and reared there their palaces and temples and altars, had already passed away at the time of the arrival of the Spaniards; and the wilderness, reclaiming the land, guarded their deserted palaces from the spoiling hand of the conqueror.

Who these people were, where they came from, what their achievement was, and what ultimately befell them,—these are questions that cannot be answered at present. And yet I know of no set of questions that more answers have been given to than this very same set of questions. According to some, as you all know, the lost ten tribes of Israel travelled all the way across Asia, past the Behring Sea on the ice, until they found a suitable habitat in America. According to others, the Eastern Asiatics did something of the same sort. According to others, the Phœnicians included America in their colonial policy. And according to others still, the Irish, in some indefinite, remote past, anticipated their posterity by emigrating to America. But all of such theories are equally worthless, and no satisfactory answer can at present be made.

As to what became of the people in the end, of course it is easy to think of pestilence, of earthquake, of famine, of civil war; but it is very hard, indeed, in the light of the facts which we can glean from our investigations, to give an answer that is at all satisfactory. It would seem, however, that a remnant of this same ancient people exists in the tribes that inhabit the Central America of to-day, but by no means pure. If they are, as we believe, the remnant of that old race, they are mixed with other races, barbarians, very likely, who lived on the outskirts of the ancient civilization of Central America until its downfall. They all speak dialects pertaining to a common stock language, called Maya, the language spoken by the ancient people of Central America.

The name Maya, then, is the name by which that ancient people is called. I have not time to discuss the name with you, to trace its origin, and show the reasons why it is applied; but it is the name which is appropriate to that people and to the remains that they have left behind them. These remains are found, as I said, scattered all the way from the Isthmus of Tehuantepec to the Isthmus of Panama. They are most abundant on the peninsula of Yucatan and in Guatemala. The latter is the most northerly of the Central American states. It occupies the whole of the Mexican boundary, with the exception of the short piece occupied by the north end of British Honduras which takes a slice off the east coast of Guatemala. South of Guatemala and British Honduras comes the republic of Honduras; next to that is Nicaragua, and between that and the Isthmus lies the republic of Costa Rica. The ruins which we are considering are found all the way from the southern states of Mexico to the boundaries between Honduras and Nicaragua; and there the land becomes quite narrow, so it may be said that the greater part of Central America was at one time occupied by this great nation.

It was to explore these ruined cities that expeditions were organized and sent out by the Peabody Museum, Harvard University, the first one leaving Cambridge in the fall of 1890. The collections brought to Cambridge, which are very extensive, represent almost all of the important groups of ruins, and though they can convey no adequate idea of the ruins to which they pertain, they are sufficient to show that the people attained in certain lines of industry a marvellous skill, and that among the ancient peoples of the earth they occupy a very high position. The results of these explorations will ultimately be published in the memoirs of the Peabody Museum. Two memoirs have appeared in which outlines of the work accomplished have been given, and another one is going through the press now, in which certain parts of the work are taken up and discussed in detail and deductions made.

Most of the work done by these expeditions from Cambridge has been carried on in the old city of Copan, which is on the border between the republic of Guatemala and the republic of Honduras; it is really in Honduras, midway between the two oceans, at an elevation of about two thousand feet, but you have to climb mountains five thousand feet high in order to get there from either side.

The first account given of Copan was given by the American traveller Stevens, in 1839. Stevens was an American special diplomatic agent, sent by the President of the United States to the republic of Central America. At that time Central America was one republic. Mr. Stevens arrived there at a time when the dissolution of that republic had begun, when Carrera with his army of undisciplined Indians was overrunning the country, compelling the government to take refuge first in one state then in another. Stevens, arriving there in that situation, set out in pursuit of the government to which he was accredited, and during this chase he stumbled on the ruins of Copan. Stevens was so impressed with the few glimpses of the ruins that he had that he persuaded his companion, Mr. Catherwood, who was an artist, to remain, while he pressed on in pursuit of the government, and make some drawings of the monuments. Catherwood's pencil drawings of the principal monuments that were at that time visible, executed with marvellous fidelity, were included in Stevens's book, which was published simultaneously in England and America in 1840, and while it caused considerable interest and was widely read simply because of the charm of Stevens's narrative, his account of the ruins met with general discredit and misbelief. Nobody seemed to be able to believe that such remains lay buried in the forests of Central America.

In 1885 the English traveller, Alfred Maudslay, of London, having to go to the tropics for the sake of his health, and having read Stevens's work and been interested in it, made up his mind to follow Stevens's trail and find out whether such a ruined city existed or not. He was successful in finding Copan, where he remained long enough to make photographs and moulds of the principal monuments. It was only then that a general interest was awakened in the scientific world concerning the ancient cities of Central America.

Without entering into any detailed description of the ruins, which would necessarily be incomplete and rather perfunctory, I will endeavor to give you some of the impressions made on my mind while I sojourned among the ruins of Copan. When the first expedition from the Peabody Museum (Owens and Saville) arrived, in 1890, the ruins were found buried in an impenetrable thicket, which had obliterated all traces of Maudslay's clearings. Roads had to be cut in order to reach them, and then clearings had to be made in the dense tropical jungle, in which one is so oppressed that it seems impossible at times to breathe. It was not until several months had been spent in the cutting of the forests and clearing the ground that any conception at all could be had of the appearance of the ruins and their general character.

After the forest was cleared off, the first thing that became apparent was the evident strength and magnitude of the structures, and one began to realize the enormous time-element which they represent, not only by the ages during which they have stood, attaining an antiquity which makes the memory of man seem insignificant, but by reason of the long, laborious process, the outcome of which was the mass of architectural marvels, with its extraordinary elaboration of ornament, its intricacy of design, where the strange interweaving of unintelligible motives baffles the eye and the imagination as well. When we consider that all this work was executed with no better tools than flint chisels, it really seems marvellous, and brings home to us the feeling that the old Mayas were a people of infinite patience and perseverance, in addition to being possessed of an artistic instinct of a high order. Everything pertaining to these ruins is so strange and unfamiliar that the experience is like what one might expect upon being transported to a different planet where there are beings with an entirely different set of mental faculties, who have different ways of thinking and acting, and living a life entirely different from ours. And yet once in a while I would come across a little touch of human nature, such as evidence of the love of personal adornment, which reminded me that, after all, this human race is all akin and that the distinctions between its different branches are not so very wide. Still the pervading spirit of the place was one of profound mystery, a mystery made all the more impressive by the silence and desolation that surround these perishing relics of a barbaric pomp and splendor unsurpassed anywhere in the world's history.

The problem suggested to the mind on inspection of the ruins is the problem of the inscriptions. There stand the monuments and the temples, on whose walls are carved what, in all probability, is the history of the people, and yet we cannot read it. Several attempts have been made to work out a key to this system of hieroglyphics, but none of them have been of any use whatever. Some have tried it by analogy with the Egyptian system, but without satisfactory results. Others have tried to assign a phonetic value to each element of the hieroglyphics, according to a suggestion in the form, or according to their own fancy, but the so-called keys obtained in this way were found to be inapplicable except by the inventor. Numerous other very ingenious keys have been worked out, and we have heard it announced from time to time very emphatically that a key had been discovered which will unravel the whole mystery of the inscriptions. The only way in which a clew can be arrived at is by a careful comparison of one inscription with another, and of one element in each inscription with the other elements. We have, as a starting-point in this problem of the hieroglyphics, the records handed down by the early missionaries in Yucatan, who, while they destroyed the documents which they found among the natives, have given us the meaning of twenty symbols found among the Indians still living. These twenty characters were the names of the days in the month, and it has now been proved that these characters in use among the half-wild tribes, that Bishop Landa and his contemporaries found in Yucatan, were the same signs used in Copan and all the other ancient cities. Taking that as a starting-point, this much progress has been made: that we are enabled to read the inscriptions so far as they apply to chronological records, and sometimes, even where the matter concerns astronomical calculations, it has been interpreted, although there is not a sign or inscription of which it can be said that we know the meaning of the whole.

Every long inscription begins with a date, recorded by means of a chronological system that was really remarkable in its perfection. The length of the year in the annual calendar was three hundred and sixty-five days, and it appears that the astronomers were perfectly well aware of the extra fraction of a day which had to be accounted for in order to make the count right. That was the annual calendar. The chronological calendar was different. In keeping chronological records extending over long periods of time they adopted a different year, a year of three hundred and sixty days. This must in the first place have led to some confusion, having an annual calendar of three hundred and sixty-five days, and in the chronological calendar another period of three hundred and sixty days, and it would appear as though the astronomers and mathematicians were very much exercised over the problems involved, and in trying to work the two systems and get them to work harmoniously, they incidentally worked out a really remarkable system of mathematics, and at the same time developed, to an astonishing degree, the science of astronomy.

The time-count proceeded by periods of three hundred and sixty days up to a period of seven thousand two hundred days. After that there was a longer period of one hundred and forty-four thousand days, according to the vigesimal system, which is at the basis of the numeral system of the Mayas as well as that of the Aztecs. It is now generally recognized that there was another much longer period, which would give to the time-count an extraordinary length and place the starting-point far in the past, but as there is still some doubt as to the part played by this period I will not discuss it any further. It is enough to say that the earliest and latest dates, recording, as we believe, the dates on which certain monuments were erected, are about two hundred years apart. This period would of course correspond to the latest period of constructive activity, and is a small part of the period during which the city flourished. There are two reasons for this conclusion,—first, the characters on the earliest monument are as fully developed as those on the latest, and secondly, our excavations have revealed the fact that below the foundations of these later monuments are the remains of older and cruder structures, and there is evidence that these older structures are not in one layer only, but in several layers, corresponding to different periods or eras of occupation. It should be mentioned that the Maya chronology has not yet been brought into touch with our own, so that the dates give us the relative and not the actual age of monuments.

I have not time to enter into a more detailed description of the inscriptions and the system of hieroglyphics, and the attempts that have been made to interpret them. But I may say this: that the results already attained give good ground for belief that in the near future the whole system will be worked out, and that there will be no more mystery about the inscriptions of Central America.

Next to the inscriptions on the walls and monuments, it was the tombs that seemed at first to offer the greatest chance of furnishing material that would help to illustrate the life and customs of the people, their religious beliefs, and their social organization. It was also hoped that manuscripts might be found in the tombs, for it is well known that the Mayas had books printed by hand on paper made from maguey fibre. As a matter of fact, the tombs contained little except the teeth of the occupant. In almost all of them there were beautiful funeral urns, which, however, contained nothing. There were also bone carvings and jade ornaments which had evidently been worn by the dead at the time of burial. If manuscripts were placed in the tombs they have entirely decomposed.

Perhaps the most interesting ethnological fact revealed by the contents of the tombs was that furnished by the teeth. It is a curious thing that the teeth, which in the living body are the part most subject to decay, last, after we are all dead, when every other trace of the body has disappeared. The interesting thing about the teeth from the tombs at Copan is the fact that they are set with jewels. Now, there is abundance of evidence that the inhabitants of Copan were vain and fond of personal adornment. What you find on the walls of the temples and on the sculptured monuments is not, as might be expected, intended to depict the exploits of warriors or the pomp and circumstance of war. There are no processions with martial accoutrements or the spoils of victory. Such things seemed to be unknown and foreign to their interests, and in place of this you see personages posing with apparently no other object than to display their wonderful ornaments and richly worked attire. So, also, we see by the remains in the tombs that they not only set jewels in their teeth, but they also filed the front teeth according to some fashion prevailing among them.

It is altogether likely that this practice of setting jewels in the teeth (it was done with consummate skill) led to a profession similar to the profession of dentistry. I am morally certain that in that old city there were schools of philosophy and schools of science, and there is no reason for supposing that they did not have schools of medicine and surgery.

The social and political conditions of the modern Honduranians present a striking contrast to the conditions prevailing among their ancient forerunners.

The ethnological aspect of these people affords an interesting experiment in the mixing of races. It is only in a few of the more remote corners of the country that Indians of pure blood are found, and their numbers are small. There are no longer any Spaniards of pure blood. With the exception of the few Indians the population is made up of a mixture of Spanish and Indian, together with a strong Moorish element and an infusion of negro blood. Those optimistic dreamers who are wont to look with complacency on these United States as the melting-pot where the amalgamation of the most diverse ethnical elements is to be accomplished for the improvement of the human race, might do well to study the conditions in those countries where the process of amalgamation has already been accomplished. Governments change with a rapidity which baffles the historian. The warlike attributes for which we looked in vain among the sculptured monuments of Copan are conspicuously present. Liberty is a word that is stamped upon the coins and postage, and printed in big letters on the flag. Individual enterprise is confined to one branch of industry,—the making of revolutions. A man is spoken of as a good revolutionist as a man here is said to be a good debater or a good party leader. It is the only profitable branch of industry, because, if a man is a successful planter or miner, he may lose his labor and his property in the next revolution, whereas if he is a successful revolutionist, the other man does the losing. The majority of the people who really have no voice whatever in the government, except when they are called upon to serve as "volunteers," are a simple-minded and hospitable people, a people with few wants and these easily satisfied. Nature is generous and supplies all their wants with but little labor. One hardly knows whether to envy such people or to pity them. It is true that they are subject to great afflictions to which we are practically immune by reason of our superior knowledge, our science, our social conditions, and physical comforts, and yet it must be admitted that they are happier than we are. What suffering they have is entirely physical. Above all, they enlist the sympathy of the traveller by reason of their simple faith in his power over pain and disease. According to their firm belief, all foreigners are doctors, and a doctor is supposed to be able to dispose of all forms of physical infirmity as readily as the priest disposes of their sins in consideration of a piece of four reals. They seem to have lost faith in their own quack doctors, probably because they have learned too much about their methods, but when the foreigner fails to fulfil their expectations, it is not because he cannot, but simply because he does not choose, hence the whole problem consists in prevailing upon him either by prayers and entreaties or else by offers of material reward. The more unwilling you are to practise upon their ignorance and credulity the more earnest and pitiful do their entreaties and offers become. The traveller who takes some trouble to minister to the sick along his way, is no loser for pains, because in return for a dose of medicine for a sick brother, sister, wife, husband, or child, all that they possess is at his disposal. I am glad to be able to say that I do not think I ever did them any harm by my practice among them, while I am sure that I was often able to do much good. The diseases to which they are most subject are fevers, which are owing more to their habits of life than to the climatic conditions. Usually all they need is a little soap and water, but after a long series of attempts to introduce this commodity among them I gave it up. It was too pitiful to see the look of disappointment on a woman's face, when, in answer to her appeal for "remedios" she received a piece of soap and was told to go and wash herself, whereas the genuine satisfaction with which she received a packet of powders of any sort was a real pleasure. Most of my operations were performed when called upon to extract a tooth, which happened not so frequently as one might think, considering that no care whatever is taken of the teeth. As a rule, the people of all classes have good teeth, which stay by them through life. Whether this is due to racial characteristics, to climatic conditions, or to personal habits I am unable to say. One thing is worth noticing in this connection,—that all classes and both sexes of all ages use tobacco to a great extent. Nothing is more common than to see a child that has not yet learned to walk sitting with great composure on the ground, occupied with a big cigar, and gravely contemplating the world through a cloud of tobacco smoke; and old age looks out upon the same world through the same soothing medium.

